# Kutane und nichtkutane Erkrankungen durch *Mycoplasma pneumoniae* bei Kindern

**DOI:** 10.1111/ddg.70052x

**Published:** 2026-04-08

**Authors:** Hanna Lindemann, Maximilian David Mauritz, Victoria Rehmann, Michael Paulussen, Jorge Frank, Malik Aydin

**Affiliations:** ^1^ Klinik für Dermatologie und Allergologie, Universitätsspital Basel Basel Schweiz; ^2^ Lehrstuhl für Pädiatrie, Vestische Kinder‐ und Jugendklinik Datteln, Universität Witten/Herdecke Datteln Deutschland; ^3^ Klinik für Dermatologie, Venerologie und Allergologie, Universitätsmedizin Göttingen Göttingen Deutschland; ^4^ Translationale Medizin mit Schwerpunkt Pädiatrische Infektiologie, Department für Humanmedizin Fakultät für Gesundheit, Universität Witten/Herdecke Witten Deutschland; ^5^ Virologie und Mikrobiologie, Zentrum für Biomedizinische Ausbildung und Forschung (ZBAF), Department für Humanmedizin Fakultät für Gesundheit, Universität Witten/Herdecke Witten Deutschland; ^6^ Institut für Medizinische Labordiagnostik, Zentrum für Forschung in der Klinischen Medizin (ZFKM) Helios Universitätsklinikum Wuppertal, Universität Witten/Herdecke Wuppertal Deutschland

**Keywords:** Erythema exsudativum multiforme, Erythema nodosum, Mycoplasma‐induced rash and mucositis, Mycoplasma pneumoniae, Stevens‐Johnson‐Syndrom, Toxische epidermale Nekrolyse, Erythema multiforme, Erythema nodosum, Mycoplasma‐induced rash and mucositis, Mycoplasma pneumoniae, Stevens‐Johnson syndrome, Toxic epidermal necrolysis

## Abstract

*Mycoplasma pneumoniae* (MP) ist ein häufiger Erreger verschiedener Infektionen bei Kindern und Jugendlichen, die vor allem die Atemwege betreffen. Neben atypischen Pneumonien kann MP auch zu extrapulmonalen Manifestationen führen, darunter mukokutane, hämatologische, neurologische, kardiale und gastrointestinale Symptome. Diese extrapulmonalen Manifestationen werden oft übersehen, was die Diagnose und Behandlung erschwert. Hier beschreiben wir das Spektrum der durch MP verursachten kutanen und nichtkutanen Erkrankungen im Kindesalter und konzentrieren uns dabei auf die Pathophysiologie, die klinischen Merkmale und die Behandlung mukokutaner Manifestationen.

## EINLEITUNG

*Mycoplasma pneumoniae* (MP) ist ein kleines, pleomorphes Bakterium aus der Klasse der Mollicutes. Es wird in erster Linie durch Tröpfcheninfektion übertragen und ist eine häufige Ursache ambulant erworbener Pneumonien, insbesondere bei Kindern im schulpflichtigen Alter. Die höchste Inzidenz findet sich im Spätsommer und Herbst, wobei es alle 3 bis 7 Jahre zu Epidemien kommt.^1^ Nach dem Ende der Maßnahmen gegen COVID‐19 war ein Anstieg der Infektionen zu beobachten, was die Bedeutung dieses Erregers unterstreicht.^2^

Nach der Übertragung haftet sich MP an das Flimmerepithel der Atemwege und ermöglicht so die Kolonisierung. Eine asymptomatische Besiedlung kann selbst unter antimikrobieller Therapie über Wochen bis Monate persistieren.^3^ Nach der Kolonisierung setzt MP Zytotoxine frei, die das Gewebe des Wirts befallen und sowohl lokale als auch systemische Entzündungsreaktionen auslösen können. Dieser Prozess führt zu einer Vielzahl von Krankheitssymptomen, die überwiegend auf indirekte immunvermittelte Effekte zurückzuführen sind.^1^, ^4^

## DIAGNOSE

Die Diagnose basiert auf der klinischen Untersuchung und Expositionsanamnese, unterstützt durch Laboruntersuchungen. Spezies‐spezifische Untersuchungen mittels Polymerase‐Kettenreaktion (PCR) haben eine hohe Sensitivität, können jedoch nicht zuverlässig zwischen Kolonisierung und aktiver Infektion unterscheiden.^3^, ^5^ Serologische Untersuchungen werden häufig eingesetzt, wobei IgM‐Antikörper in der Regel in frühen Phasen freigesetzt werden, gefolgt durch den Nachweis von IgG‐Antikörpern 3 bis 4 Wochen nach der Infektion.^5^ Der Nachweis MP‐spezifischer IgM‐sezernierender Zellen im peripheren Blut ist der stärkste Hinweis auf eine akute Erkrankung. Dennoch wurde diese Methode auf Grund des selbstlimitierenden Krankheitsverlaufs noch nicht im klinischen Alltag implementiert.^6^ Darüber hinaus werden Kulturen aufgrund des schwierigen und langsamen Wachstums der Bakterien nur selten durchgeführt.

Je nach klinischem Bild können zur Diagnosestellung bei Verdacht auf eine Pneumonie auch bildgebende Verfahren wie Röntgenaufnahmen des Thorax, herangezogen werden.^7^, ^8^

## KLINISCHE MANIFESTATION

### Nichtkutane Erkrankungen

Eine Pneumonie ist die häufigste durch MP hervorgerufene klinische Manifestation bei Kindern im schulpflichtigen Alter. Nach unproduktivem Husten leiden die Patienten in der Regel unter leichtem Fieber, Kopfschmerzen und Unwohlsein.^7^ Die radiologischen Befunde sind variabel und meistens unspezifisch. Sie beinhalten eine ein‐ oder beidseitige Bronchopneumonie im Unterlappen oder perihilär mit retikulonodulärer Eintrübung.^8^ Extrapulmonale Manifestationen können ebenfalls auftreten. So kann bei etwa 50% der Patienten eine Kälteagglutinin‐Reaktion beobachtet werden, die durch gegen die Erythrozytenmembran gerichtete IgM‐Antikörper hervorgerufen wird.^9^ Eine klinisch signifikante Hämolyse kann Patienten mit chronischen Erkrankungen, einschließlich einer Sichelzellanämie, betreffen. Des Weiteren entwickeln etwa 5% der hospitalisierten pädiatrischen Patienten Symptome des zentralen Nervensystems, hierunter eine Meningoenzephalitis und eine akute disseminierte Enzephalomyelitis. Ebenso können gastrointestinale Symptome auftreten, insbesondere Bauchschmerzen, Erbrechen und Diarrhoe. Nur selten werden rheumatologische, kardiale und renale Erkrankungen bei pädiatrischen Patienten beobachtet.^10^

Während Atemwegsinfektionen das Hauptmerkmal einer MP‐Infektion sind, können lipidassoziierte Membranproteine potenziell auch als Superantigene fungieren.^11^ Die systemische Immunantwort korreliert mit dem Schweregrad der Atemwegserkrankung und kann verschiedene extrapulmonale Manifestationen hervorrufen.^12^, ^13^

Unter biologischen Gesichtspunkten weist MP eine einzigartige Struktur ohne echte Zellwand auf, was zur physiologischen Resistenz gegenüber β‐Laktam‐Antibiotika führt. Im Gegensatz hierzu sind Makrolide, Tetracycline und Fluorchinolone die Antibiotika der Wahl. Bei Kindern sind Fluorchinolone jedoch gegenüber anderen Wirkstoffen auf Grund einer vergleichbaren Effizienz und des Risikos irreversibler Knorpelschäden nicht in erster Linie indiziert. Da die meisten durch MP verursachten Atemwegsinfektionen selbstlimitierend sind, ist bei milden Erkrankungsverläufen zuwartende Haltung angemessen, und eine Antibiotikabehandlung sollte Patienten mit schwerem oder langwierigem Verlauf vorbehalten bleiben.^1^ Zukünftige Studien müssen zeigen, ob Makrolide überhaupt einen Einfluss auf den Verlauf einer durch MP verursachten ambulant erworbenen Pneumonie haben.^10^ Tabelle 1 fasst die häufigsten nicht‐kutanen Manifestationen im Zusammenhang mit MP bei Kindern zusammen.

**TABELLE 1:** Die häufigsten mit einer *Mycoplasma‐pneumoniae*‐Infektion assoziierten nichtkutanen Erkrankungen bei Kindern.

Erkrankung(en)	Organbeteiligung/‐erkrankung(en)	Klinische Symptome/Charakteristika
*Mycoplasma pneumoniae*	Infektion der unteren Atemwege	Fieber, unproduktiver Husten; Fatigue; Dyspnoe und/oder Kopfschmerzen
Pharyngitis Asthma	Andere Atemwegserkrankungen	Halsentzündung; Schnupfen; Kopfschmerzen; Ohrenschmerzen; verlängertes Husten oder Giemen
Sekundäres Kälteagglutinin‐Syndrom	Hämatologische Erkrankungen	Klinisch unrelevante Hämolyse oder schwere Hämolyse bei Risikopatienten mit beispielsweise Sichelzellanämie
Meningoenzephalitis Akute disseminierte Enzephalomyelitis Transverse Myelitis Zerebelläre Ataxie Guillain‐Barré‐Syndrom Zerebraler/Zerebellärer Infarkt Periphere Neuropathie Kraniale Nervenlähmung(en)	Erkrankungen des zentralen Nervensystems	Neurologische Symptome in Korrelation mit den jeweiligen Erkrankungen; Lymphozytäre Pleozytose; erhöhte Proteinkonzentration(en) und normale Glukosekonzentration
Splenomegalie Intussuszeption Hepatomegalie Pankreatitis	Gastrointestinale Erkrankungen	Bauchschmerzen; Erbrechen; Diarrhoe
Septische oder reaktive Arthritis oder Arthralgie Rhabdomyolyse	Rheumatologische und muskuloskelettale Erkrankungen	Gelenk‐ oder Muskelschmerzen oder‐schwellung
Myokarditis Perikarderguss Intravaskuläre Thromben Morbus Kawasaki Herzversagen	Kardiovaskuläre Erkrankungen	Tachy‐/Bradykardie; Herzversagen
Sekundäre Glomerulonephritis nach Immunkomplexablagerung(en)	Renale Erkrankungen	Akute Nephritis; nephrotisches Syndrom oder akutes Nierenversagen und Proteinurie

### Kutane Erkrankungen

Hautsymptome treten bei 10% bis 25% der MP‐Infektionen auf, wobei Kinder häufiger betroffen sind als Erwachsene.^7^ Zu den Hauterscheinungen, die sowohl bei aktiven als auch bei asymptomatischen Infektionen auftreten können gehören unspezifische makulopapulöse Exantheme, *Mycoplasma‐induced rash and mucositis* (MIRM), Erythema nodosum (EN), Urtikaria, Erythema exsudativum multiforme (EEM) und Stevens‐Johnson‐Syndrom (SJS). Seltene Erkrankungen umfassen Pityriasis rosea, toxische epidermale Nekrolyse (TEN), leukozytoklastische Vaskulitis, Immunkomplex‐Vaskulitis, thrombozytopenische Purpura, subkorneale Pustulose (Sneddon‐Wilkinson‐Syndrom), Sweet‐ Syndrom, Raynaud‐Syndrom, Reiter‐Syndrom, Urtikaria‐Vaskulitis und Gianotti‐Crosti‐Syndrom (Tabelle 2).^4^, ^6^, ^10^, ^14^

**TABELLE 2:** Die häufigsten mit einer *Mycoplasma‐pneumoniae*‐Infektion assoziierten Hauterkrankungen bei Kindern.

Erkrankung	Häufigkeit aller berichteten Fälle mit einer *M.‐pneumoniae*‐Infektion	Hautmanifestationen	Verteilung	Schleimhautbeteiligung	Systemische Beteiligung
Unspezifisches makulopapulöses Exanthem	8–33%	Makulopapulöses Exanthem	Oberkörper und Extremitäten ± Palmae und Plantae	Fehlt üblicherweise oder tritt selten auf; nur in Einzelfällen beschrieben	Üblicherweise mild oder fehlend
*Mycoplasma‐induced rash and mucositis*	13 Fälle	Mild ausgeprägtes polymorphes Exanthem oder keine Hautveränderungen Typischerweise vesikulobullöse oder atypische Schießscheibenläsionen	Vorwiegend Schleimhautbeteiligung Abschilferung/Ablösung von < 10% der Hautoberfläche	Schwer; ≥ 2 Körperregion (beispielsweise oral, okulär, anogenital, nasal)	Üblicherweise moderat, Fieber, Unwohlsein
Erythema nodosum	8%	Schmerzhafte erythematöse Knoten	Typischerweise an den Unterschenkeln Weniger häufig and den Unterarmen und Oberschenkeln	Fehlt	Üblicherweise mild, Fieber, Arthralgie, Unwohlsein
Urtikaria	7%	Anuläre Plaques ± Schwellung des Gesichts, der Hände oder Füße (Angioödeme)	Disseminiert Einzeleffloreszenzen haben einen vorübergehenden Charakter (< 24 h)	Fehlt	Üblicherweise mild oder fehlend
Erythema exsudativum multiforme minus	3,6–7%	Typische Schießscheibenläsionen ± Papulose‐atypische Schießscheibenläsionen	Extremitäten	Fehlt oder mild ausgeprägt	Üblicherweise mild oder fehlend
Erythema exsudativum multiforme majus		Typische Schießscheibenläsionen ± Papulose‐atypische Schießscheibenläsionen	Extremitäten und Gesicht	Schwer	Üblicherweise mild, Fieber, Arthralgie
Stevens‐Johnson‐Syndrom	1–5%	Typische Schießscheibenläsionen mit Ablösung der Epidermis und Erosionen Bullöse Hautveränderungen	Isolierte Effloreszenzen mit Konfluenz am Stamm und im Gesicht Abschilferung/Ablösung von < 10% der Hautoberfläche	Schwer	Üblicherweise schwer; Fieber, Lymphadenopathie, Hepatitis, Zytopenie

### Unspezifisches makulopapulöses Exanthem

Die häufigsten MP‐assoziierten Hautmanifestationen sind unspezifische makulopapulöse Exantheme. In einer prospektiven Studie an 152 Kindern mit ambulant erworbenen Pneumonien wiesen 22,7% der Patienten mit mittels PCR‐bestätigtem MP‐Nachweis und spezifischen IgM‐sezernierenden Zellen mukokutane Veränderungen auf. Juckende Hautausschläge entwickeln sich häufig im frühen Stadium der Erkrankung und manifestieren sich typischerweise als kleine rote Maculae oder flache Plaques, die große Körperareale, einschließlich der Handflächen, Fußsohlen und Schleimhäute, betreffen können. Es handelt sich in der Regel um innerhalb von 2 bis 22 Tagen selbstlimitierende Exantheme, die meist gut auf symptomatische Behandlung mit Antihistaminika und topischen Glukokortikosteroiden ansprechen.^4^

Von entscheidender Bedeutung ist die Differenzierung zwischen MP‐induzierten und arzneimittelinduzierten Exanthemen. In einer retrospektiven Analyse von 81 Patienten mit serologisch bestätigter MP‐Infektion zeigte sich, dass 18,5% Hautausschläge entwickelt hatten, wobei 72% zwei oder mehr Antibiotika erhalten hatten, vorwiegend Ampicillin oder andere Penicilline, was auf arzneimittelbedingte Ursachen hindeutet.^15^

### Mycoplasma‐induced rash and mucositis

Im Jahr 2015 beschrieben Canavan et al. die MIRM erstmals als eigenständige Erkrankung, von der vor allem Jungen im vorpubertären Alter (Durchschnittsalter 11,9 Jahre) betroffen sind.^16^ Die Erkrankung ist gekennzeichnet durch eine Mukositis an mindestens zwei Körperregionen (oral, okulär und/oder genital) mit limitierter Hautbeteiligung (< 10% der Körperoberfläche), die sich häufig in Form weniger vesikulobullöser oder schießscheibenartiger Hautveränderungen manifestiert. Systemische Symptome können Fieber, Husten und pathologische klinisch‐ oder radiologisch‐pulmonologische Befunde umfassen. Eine MP‐Infektion kann durch erhöhte IgM‐Antikörpertiter oder eine PCR‐Untersuchung bestätigt werden.^16^

Die Abgrenzung der MIRM als von EEM und SJS zu unterscheidende Entität hat zu einer anhaltenden Debatte geführt. Klinisch weist die MIRM‐Überschneidungen mit dem EEM major und dem SJS auf, hierunter Schleimhautbeteiligung und Schießscheibenläsionen. Auch histopathologische Untersuchungen konnten keine überzeugenden Unterschiede zwischen diesen Erkrankungen aufzeigen, was ihre Unterscheidung noch weiter erschwert.^16^, ^17^

Schleimhautbeteiligungen können Untersuchungen durch Augenärzte, Hals‐Nasen‐Ohren‐Ärzte, Gastroenterologen oder Urologen erforderlich machen. Obwohl es keine Behandlungsrichtlinien gibt, können frühzeitige Antibiotikagabe und unterstützende Maßnahmen, einschließlich Schmerzkontrolle, Schleimhautpflege und Flüssigkeitsmanagement hilfreich sein. Die effektive Behandlung schmerzhafter Schleimhautveränderungen ist unerlässlich, um Sekundärinfektionen vorzubeugen.

Bei schwerer Schleimhautbeteiligung kann die intravenöse Gabe von Immunglobulinen hilfreich sein, eine schwere Hautbeteiligung die Einbeziehung spezialisierter Verbrennungszentren erforderlich machen.^16^, ^17^

Jüngste Erkenntnisse deuten darauf hin, dass auch in Assoziation mit anderen Infektionserregern gleichartige mukokutane Symptome auftreten können, was zur Verwendung des umfassenderen Terms reaktive infektiöse mukokutane Eruption, im Englischen *reactive infectious mucocutaneous reaction* (RIME), geführt hat.^18^

### Erythema nodosum

Das EN ist bei Kindern selten und durch schmerzhafte rote Knoten, vornehmlich an den unteren Extremitäten, charakterisiert.

In einer Studie wurden MP‐Infektionen durch serologische Untersuchungen bei 3 von 35 Kindern detektiert (8,6%). Interessanterweise war einer dieser Patienten asymptomatisch.^19^ In einer zweiten Studie fand sich MP auf Basis eines serologischen IgM‐Nachweises bei vier von 39 Patienten, wobei die meisten symptomatisch waren. In zwei Fallberichten konnten Doppelinfektionen durch MP und andere Erreger nachgewiesen werden.^20^

Berücksichtigt man die niedrige Detektionsrate, ist eine routinemäßige MP‐Untersuchung nicht empfehlenswert, vor allem nicht bei Fehlen von Atembeschwerden. Hingegen erscheinen zielgerichtete derartige Untersuchungen während einer MP‐Epidemie oder bei gleichzeitig bestehenden Atemwegsbeschwerden angemessen. Obwohl sich das EN üblicherweise innerhalb weniger Wochen von selbst zurückbildet, wurde die erfolgreiche Behandlung mit Makrolid‐Antibiotika gezeigt, die möglicherweise das Wiederholungsrisiko reduziert.^4^, ^10^

### Urtikaria

Eine MP‐assoziierte Urtikaria tritt vor allem bei Kindern auf. In einer Studie, die 65 Kinder mit Urtikaria umfasste, wiesen 21 Studienteilnehmer (32%) MP IgM‐Antikörper und/oder einen positiven Schnelltest auf Kälteagglutinine auf.^20^ Obwohl die klinischen Symptome allein für den Nachweis eines kausalen Zusammenhangs nicht ausreichen, ist bei Kindern mit Urtikaria und Atemwegsbeschwerden eine Untersuchung auf MP ratsam. Bei solchen Patienten kann eine Antibiotikatherapie dazu beitragen, sowohl die Dauer der Urtikaria als auch der Atemwegsbeschwerden zu verkürzen.^4^, ^21^

### Erythema exsudativum multiforme, Stevens‐Johnson‐Syndrom und toxische epidermale Nekrolyse

EEM, SJS und TEN spiegeln ein Spektrum mukokutaner Erkrankungen wieder, die eng miteinander in Zusammenhang stehen, sich jedoch hinsichtlich ihrer klinischen Manifestation und dem Schweregrad unterscheiden.

**ABBILDUNG 1:**
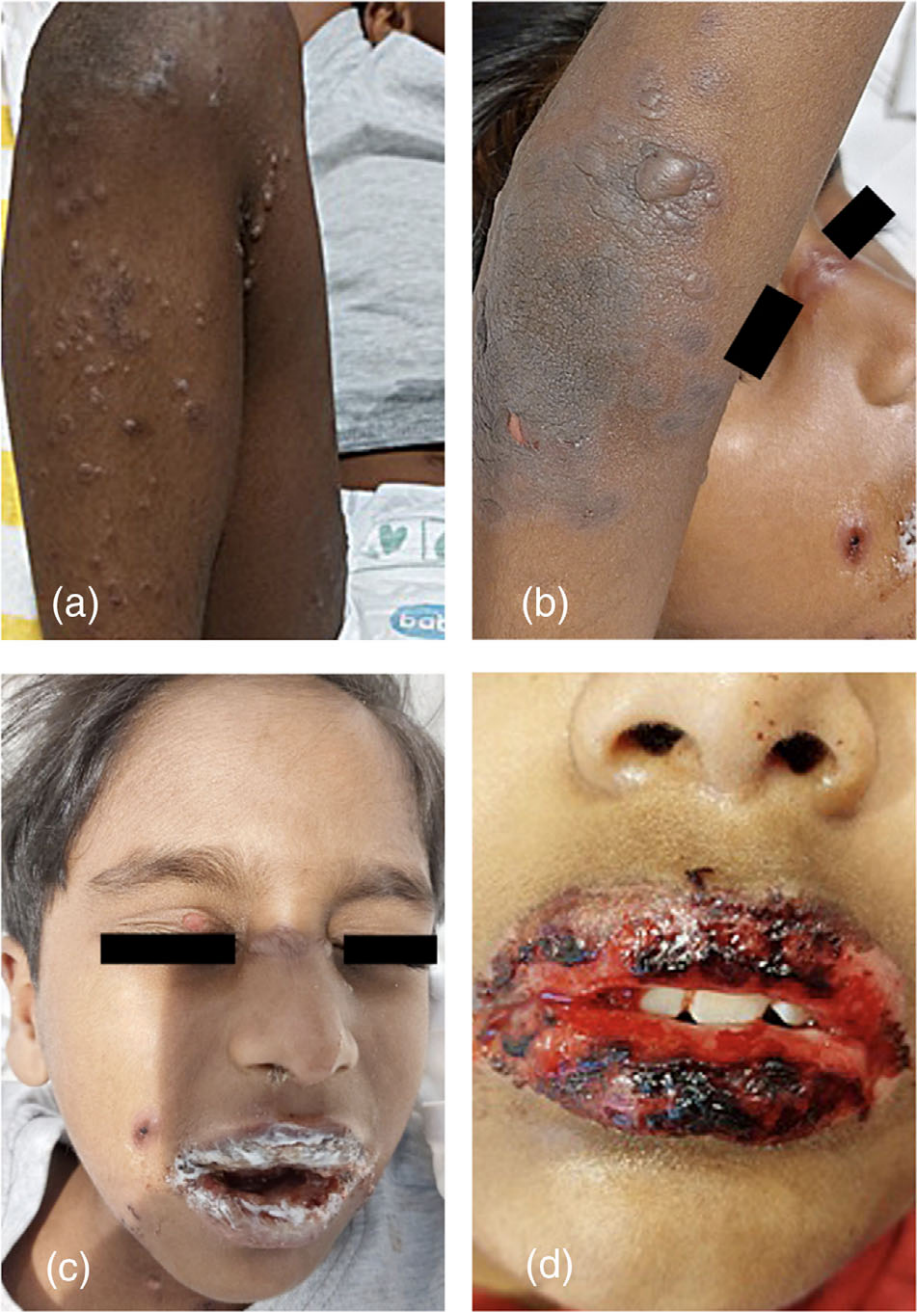
Fünfjähriger Junge mit (a) solitären bis aggregiert stehenden Papeln, Bläschen und Blasen auf teils erythematöser und teils unveränderter Haut mit partieller Kokardenformation an der rechten unteren Extremität; (b) solitären Bläschen, Blasen und Erosionen auf erythematöser Haut mit partieller Kokardenformation an der rechten oberen Extremität; (c) konfluierenden und partiell hämorrhagischen Erosionen und Krusten an den Lippen, Krusten am rechten Naseneingang, perioralen Papeln und Erosionen auf teils erythematösem Hautgrund sowie fazialen Milien; (d) zehnjähriger Junge mit konfluierenden hämorrhagischen Erosionen und Krusten an den Lippen (Die Genehmigung zur Vervielfältigung von Bildmaterial aus Lindemann H et al., Kinderärztliche Praxis 96, 29–33, 2025 wurde von MedTriX, Inc., Unter den Eichen 5, 65195 Wiesbaden, Deutschland erteilt. Das Urheberrecht für das Bildmaterial liegt bei der Dermanostic GmbH, Merscheider Straße 1, 42699 Solingen, Deutschland).

Das EEM wird in eine Minor‐Form (ohne Schleimhautbeteiligung) und eine Major‐Form (mit Schleimhautbeteiligung) unterteilt. Eine aktuelle Studie zeigte, dass MP der zweithäufigste Auslöser eines EEMs bei Kindern ist. Der Erreger ist für etwa 7% der Fälle verantwortlich, die mittels IgM‐ und IgG‐Serologie sowie anhand der klinischen Manifestation bestätigt werden.^22^ Klinisch finden sich multiple Schießscheibenläsionen an den Extremitäten, einschließlich der Palmae und Plantae, auch bei solchen Patienten, die über mildes Fieber und Unwohlsein hinaus nur eine minimale systemische Beteiligung aufweisen. Bei einigen Patienten kann es jedoch zu einer Schleimhautbeteiligung kommen, die eine stationäre Behandlung erforderlich macht (Abbildung 1).^14^, ^22^

Im Gegensatz hierzu tritt das SJS mit vorwiegend am Rumpf lokalisierten konfluierenden atypischen Schießscheibenläsionen mit obligatorischer Schleimhautbeteiligung auf. Beim SJS ist MP der vorherrschende Infektionserreger und die Ursache der Erkrankung bei etwa 22% der betroffenen Kinder.^23^

Die Überschneidung der klinischen Merkmale beim EEM und SJS ist gut bekannt, wogegen bisher der Übergang vom EEM zur TEN noch nicht nachgewiesen wurde.^24^

Gegenwärtig gibt es keine standardisierten Behandlungsprotokolle für das MP‐induzierte EEM, SJS oder die TEN. Die frühzeitige Einleitung einer systemischen Therapie mit Glukokortikosteroiden und Antibiotika kann jedoch den Krankheitsverlauf verbessern. Eine supportive Therapie wie bei der MIRM beschrieben ist unerlässlich und kann eine interdisziplinäre Zusammenarbeit erforderlich machen.^24^

## SCHLUSSFOLGERUNGEN

Infektionen durch MP können sich mit einer Vielzahl atemwegsunabhängiger Symptome und Krankheitserscheinungen manifestieren, insbesondere bei pädiatrischen Patienten. Die Zellmembran von MP kann über ortsständige inflammatorische Zytokine als Super‐Antigen fungieren und so zu einer Dysregulation des Immunsystems führen.

Die Erkennung atemwegsunabhängiger Manifestationen im Zusammenhang mit MP ist für die frühzeitige Diagnose entscheidend. Bei Kindern ist die schnelle Detektion der Infektion wichtig, da diese die Behandlung beeinflussen und potenziellen Komplikationen vorbeugen kann. Die Unterscheidung aktiver Infektionen von asymptomatischen Besiedlungen bleibt jedoch diagnostisch herausfordernd, da MP auch in den oberen Atemwegen gesunder Menschen persistieren kann. Dies unterstreicht, dass diagnostische Ergebnisse vorsichtig interpretiert werden müssen, um unnötigen Antibiotikaeinsatz zu vermeiden.

## DANKSAGUNG

Open access Veröffentlichung ermöglicht und organisiert durch Projekt DEAL.

## INTERESSENKONFLIKT

H.L. hat Vortragshonorare von Blueprint Medicines, Novartis und OrPha Swiss GmbH erhalten. J.F. erhielt finanzielle Unterstützung für die Organisation wissenschaftlicher Meetings, Symposien und Kongressreisen sowie Honorare für Beratertätigkeiten und Vorträge von AbbVie, Alnylam Pharmaceuticals, Bristol‐Myers Squibb, Clinuvel Pharmaceuticals, Genzyme, La Roche‐Posay, Lilly Deutschland GmbH, MedKomAkademie GmbH, MSD, Novartis, Orphan Europe und Shire. Er ist Senior Consultant bei Dermanostic, Inc. und Associate Partner bei medi‐login, Inc. M.D.M., V.R., M.P. und M.A. geben an, dass kein Interessenkonflikt besteht. Die Autoren erklären, dass finanzielle Interessen keinen Einfluss auf die Erstellung des Manuskripts hatten.
